# Intestinal microbiota: A promising therapeutic target for hypertension

**DOI:** 10.3389/fcvm.2022.970036

**Published:** 2022-11-15

**Authors:** Dating Sun, Hui Xiang, Jiangtao Yan, Liqun He

**Affiliations:** ^1^Department of Cardiology, Wuhan No. 1 Hospital, Wuhan Hospital of Traditional Chinese and Western Medicine, Wuhan, China; ^2^Infectious Disease Department, Chongqing University Three Gorges Hospital, Chongqing, China; ^3^Division of Cardiology, Department of Internal Medicine, Tongji Hospital, Tongji Medical College, Huazhong University of Science and Technology, Wuhan, China

**Keywords:** hypertension, intestinal microbiota, intestinal dysbiosis, SCFAs, inflammation

## Abstract

Hypertension has developed into an escalating serious global public health problem with multiple and unclear pathophysiological mechanisms. Recent studies have identified intestinal microbiota as a key perpetrator of hypertension through a variety of mechanisms. In this review, we highlight the potential roles of the intestinal microbiota and its metabolites in the development of hypertension, as well as the therapeutic potential for targeting intestinal microbiomes. We also shed light on the main limitations and challenges of the current research and suggest directions for future investigations. Finally, we discuss the development of accurate and personalized preventive and therapeutic strategies for hypotension by the modulation of intestinal microbes and metabolites.

## Introduction

Hypertension is recognized as the most prominent risk factor for cardiovascular disease (CVD) and stroke worldwide (1, 2) and leads to ~9.4 million deaths globally every year (3). In the United States, approximately half of the adult population has hypertension (4); in China, the number of adult patients with hypertension between 2012 and 2015 reached 244.5 million (5). Approximately, 50% of adults between 35 and 75 years of age have hypertension; however, less than one-third are receiving treatment, and fewer than one in 12 are in control of their blood pressure (6). In addition, there is a continuous increase in the prevalence of hypertension, which has led to the search for more effective strategies to prevent and modify the development of hypertension.

The pathogenesis and pathophysiology of hypertension are complex and unclear. Previous studies have shown that hypertension is thought to be driven by a combination of genetic and lifestyle factors, but genome-wide association studies show that only ~5% of the incidence of hypertension can be explained by genetics (7). In addition to genetic factors, the environment, diet, nervous system, and immune response have been reported as independent risk factors for hypertension (8, 9). Moreover, recent studies have shown that intestinal dysbiosis is regarded as an essential risk factor for hypertension (10, 11), which has provided a promising new therapeutic approach for hypertension (12, 13). Following the development and maturity of genome sequencing, bioinformatics, and metagenomics technologies, great progress has been made in studying intestinal microbiota. The intestinal microbiota contains more than 100 trillion microorganisms, but *Bacteroidetes, Firmicutes*, and *Actinobacteria* account for the vast majority (14, 15). The intestinal microbiota remains homeostatic, but the microbial composition varies between individuals (10). Meanwhile, the intestinal microbiota is intimately connected with many crucial organs or systems of the host, such as the brain, autonomic nervous system, bone marrow, kidney, vasculature, and immune system (16). Various factors lead to changes in the composition and positioning of microbiota, which are known as dysbiosis, and predispose patients to multiple diseases, such as gastrointestinal disease, obesity, type 2 diabetes mellitus (T2DM), non-alcoholic fatty liver disease, non-alcoholic steatohepatitis (NASH), chronic kidney disease (CKD), CVD, and hypertension (10, 17, 18). Studies have identified the association of intestinal dysbiosis with hypertension and associations of the brain–gut, kidney–gut, and microbial metabolite–host interactions in BP homeostasis mediated by the metabolic potential of the intestinal microbiota (19). In this review, the evidence supporting the role of intestinal microbiota in hypertension is summarized. Furthermore, the complex reciprocity between the intestinal microbiota and the development of hypertension and the underlying mechanisms is emphasized. Finally, the potential benefits of targeting the intestinal microbiota to regulate BP and prevent or treat hypertension are also described.

## The intestinal microbiota composition in rodent models of hypertension and human subjects with hypertension

**Figure 1:**
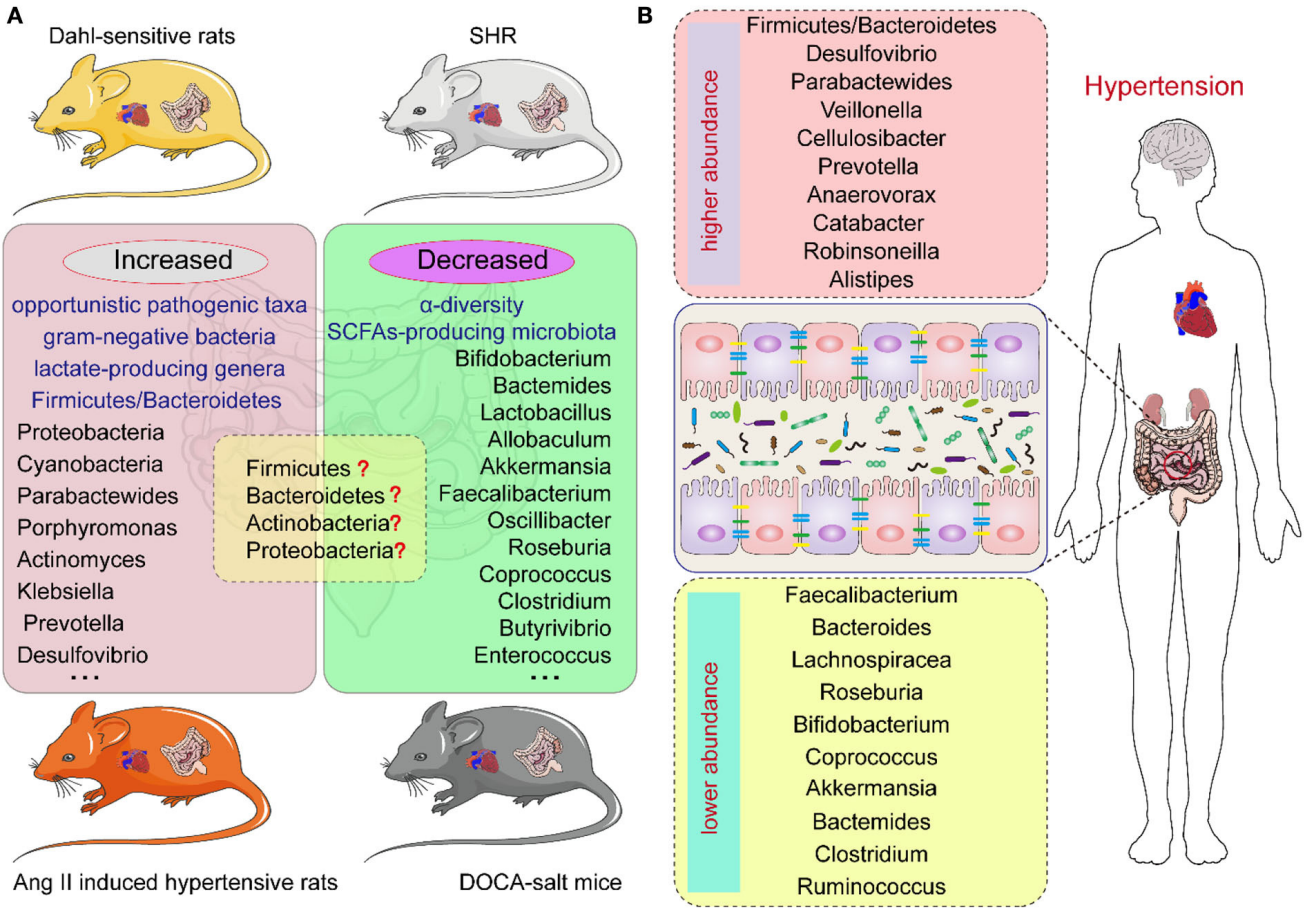
The intestinal microbiota composition in hypertension animal models and human individuals of hypertension. **(A)** The intestinal microbiota changes in different hypertension animal models. **(B)** The intestinal microbiota changes in hypertension individuals.

Studies have demonstrated a direct correlation between the gut microbiota and hypertension in both patients and animal models, including Dahl salt-sensitive rats, spontaneously hypertensive rats (SHR), angiotensin II (Ang II)-induced hypertensive rats, and deoxycorticosterone acetate (DOCA)-salt mice (20–23) (Figure 1). Bier et al. used 16S rRNA amplicon sequencing to detect the composition of the intestinal microbiota in fecal samples of Dahl salt-sensitive rats induced by a high-salt diet. They found a positive correlation between abundance and BP in six taxa, including the *Pseudomonadales* order, the *Christensenellaceae, Barnesiellaceae, Eubacteriaceae* families, and the *Erwinia* and *Anaerofustis* genera, whereas the abundance of the *Anaerostipes* genus showed a significant negative correlation with BP (24). Wilck et al. revealed that the distribution of *Lactobacillus* spp. was suppressed in high-salt diet-induced hypertensive mice, as the supplementation of *Lactobacillus* spp. in a mice model was shown to attenuate salt-sensitive hypertension, presumably by modulating the response of Th17-cells (25). In high-salt diet-induced hypertensive rats, Bier et al. found that the abundance of the *Erwinia* genus, *Christensenellaceae*, and *Corynebacteriaceae* was increased, whereas that of *Anaerostipes* was significantly reduced (24). Another study in rats with high-salt-induced hypertension showed that *Spirochaete, Actinobacteria, Firmicutes*, and *Proteobacteria* were elevated and *Verrucomicrobia* and *Bacteroidetes* were decreased (26). Yang et al. isolated the fecal DNA from SHR; mean arterial pressure, MAP: 148 ± 10 mmHg) and Wistar rats (MAP: 108 ± 2 mmHg), and found that the abundance of *Firmicutes* and *Verrucomicrobia* was significantly increased in SHR, whereas that of *Bacteroidetes* and *Actinobacteria* was reduced (27). Similarly, in SHR, Adnan et al. reported the proportion of *Firmicutes* and *Lactobacillus* was increased, but that of *Bacteroidetes, Adlercreutzia*, and *Bifidobacterium* was suppressed (28). In addition, in Wistar rats, Yan et al. reported that the high-salt diet-induced gut dysbiosis, including the reduction of beneficial *Bacteroides*, which could inhibit the production of intestinal-derived corticosterone induced by a high-salt diet through its metabolite arachidonic acid (26). Chima et al. found that Ang II-treated mice were accompanied by significant alterations in the microbiota (29). For example, consistent with other results, *Anaeroplasmataceae* increased in the Ang II-treated groups, whereas *Lachnospiraceae* decreased (29, 30). Moreover, shifts in the gut microbiome-associated metabolites, which are completely dependent on the intestinal microbiota, were observed in an Ang II-induced hypertension mouse model of hypertension (29). For example, microbiome associated- metabolites, such as 4-ethylphenylsulfate, *p*-cresol sulfate, *p*-cresol glucuronide, taurodeoxycholate, and taurodeoxycholic acid, were upregulated by Ang II (29, 31). However, metabolites such as *N,N,N*-trimethyl-5-aminovalerate, trans-4-hydroxyproline, indoleacetate, and xylose were significantly downregulated by Ang II treatment (29).

In hypertensive DOCA-salt rats, *Firmicutes* and *Lactobacillales* were found to be increased while *Sutterella, Actinobacteria*, and *Oscillospira* were reduced (32). Hsu et al. revealed that the proportion of *Akkermansia* and *Odoribacter* was increased and that of *Lactobacillus* was reduced in a model of maternal high-fructose diet-induced hypertension (33). Cold exposure has been recognized as an important risk factor for hypertension. Wang et al. analyzed the gut microbiota of rats using the 16S rDNA sequence in cold-induced hypertension and found that the abundance of *Quinella, Rothia*, and *Senegalimassilia* genera was significantly increased, but that of *Lactobacillus, Lachnospiraceae*, and *Ruminococcaceae* was decreased (34). Individuals with obstructive sleep apnea (OSA) are at increased risk for systemic hypertension; Durgan et al. established an OSA-related model of hypertensive rats by intermittent hypoxia and a high-fat diet. In this OSA-related model of hypertension, the abundance of *Lactococcus* and *Coprobacillus* was elevated and that of *Ruminococcaceae* was decreased (35). However, there are differences in the dysbiosis characteristics, including at the phylum, family, and genus levels, of different animal models of hypertension (36) (Figure 1; Table 1).

**Table 1:** Composition of intestinal microbiota in hypertensive models.

**Models**	**Method**	**Alternations in intestinal microbiota**		**References**
		**Increased**	**Decreased**	
SHR	16S rDNA sequencing	*Firmicutes, Verrucomicrobia*	*Bacteroidetes, Actinobacteria*	(27)
OSA rats	16S rRNA sequencing	*Lactococcus, Coprobacillus*	*Ruminococcaceae*	(35)
SHRSP rats	16S rRNA sequencing	*Firmicutes, Lactobacillus*	*Bacteroidetes, Adlercreutzia, Bifidobacterium*	(28)
HSD-fed rats	16S rDNA sequencing	*Parasutterella* spp.	*Lactobacillus, Oscillibacter, Pseudoflavonifractor, Clostridium, Johnsonella, Rothia*	(25)
HSD-fed rats	16S rRNA sequencing	*Erwinia genus, Christensenellaceae, Corynebacteriaceae*	*Anaerostipes*	(24)
HSD-fed rats	16S rRNA sequencing	*Spirochaete, Actinobacteria, Firmicutes, Proteobacteria*	*Verrucomicrobia, Bacteroidetes*	(26)
DOCA-salt rats	16S rDNA sequencing	*Firmicutes, Lactobacillales*	*Sutterella, Actinobacteria, Oscillospira*	(32)
high-fructose diet	Metagenomics Analysis	*Akkermansia, Odoribacter*	*Lactobacillus*	(33)
CIH rats	16S rDNA sequencing	*Quinella, Rothia*, and *Senegalimassilia* genera	*Lactobacillu, Lachnospiraceae, Ruminococcaceae*	(34)

HSD, high salt diet; SHR, spontaneously hypertensive; SHRSP, spontaneously hypertensive stroke prone rats; OSA, Obstructive sleep apnea; DOCA, deoxycorticosterone acetate; CIH, cold-induced hypertension.

Human investigations have illustrated the relationship between the composition of the intestinal microbiome and hypertension (37–40). Multiple clinical studies have demonstrated changes in the composition of intestinal microbiota in patients with hypertension (Table 2). Hypertension is usually accompanied by decreased intestinal biodiversity and dysbiosis, such as increased *Firmicutes/Bacteroidetes* ratio (48). In patients with hypertension, the bacteria which are beneficial to health conditions were diminished, including *Faecalibacterium, Bacteroides, Roseburia, Bifidobacterium, Coprococcus*, and *Butyrivibrio*, whereas *Veillonella, Prevotella*, and *Klebsiella* were increased (3, 11, 41, 49). Sun et al. examined cross-sectional associations between measures of gut microbial diversity and taxonomic composition and BP in 529 participants. The data showed that 18 genera were associated with hypertension, including *Anaerovorax, Clostridium IV, Oscillibacter*, and *Sporobacter*, and the distribution of *Veillonella* aligned with hypertensive individuals (42). Moreover, *Anaerovorax, Catabacter*, and *Robinsoneilla* were demonstrated to be positively correlated with hypertension (42). Dan et al. performed 16S amplicon sequencing of 129 fecal samples, including 62 cases with normal BP and 67 cases with high BP, and found that there were 54 differentially expressed genera between the hypertensive and normal BP groups; 18 genera were significantly more abundant in the hypertensive group, including *Acetobacteroides, Alistipes, Bacteroides, Barnesiella, Butyricimonas, Christensenella, Cosenzaea, Desulfovibrio, Dialister, Eisenbergiella, Faecalitalea, Megasphaera, Microvirgula, Mitsuokella, Parabacteroides, Proteiniborus, Clostridium sensu tricto*, and *Terrisporobacter* (43). Kim et al. analyzed 40 fecal samples from 22 hypertensive individuals and 18 normal controls and found that *Parabacteroides johnsonii, Eubacterium siraeum*, and *Alistipes finegoldii* were present at a higher abundance in patients with hypertension, and *Bacteroides thetaiotaomicron*, a butyrate-producing bacterium, was present at a lower abundance in the hypertensive group (44). Yan et al. characterized the gut microbiome in 60 patients with hypertension (BP ≥ 140/90 mmHg) and 60 sex-, age-, and body weight-matched healthy controls (BP ≤ 120/80 mmHg) by comparing fecal samples based on whole-metagenome shotgun sequencing. Yan et al. found that *Klebsiella, Clostridium, Streptococcus, Parabacteroides, Eggerthella*, and *Salmonella* were frequently distributed in the hypertensive gut compared with normal controls, whereas *Faecalibacterium, Roseburia*, and *Synergistetes* were higher in the control group than in patients with hypertension (45). Calderón-Pérez et al. reported a higher distribution of *Bacteroides coprocola, Bacteroides plebeius*, and *Lachnospiraceae* genera in individuals with hypertension, but a lower abundance of *Ruminococcaceae, Ruminococcaceae, Christensenellaceae, Faecalibacterium prausnitzii*, and *Roseburia hominis* in the hypertensive gut (46). Recently, Palmu et al. reported that 45 microbial genera were observed to be positively associated with BP indices, of which 27 belong to the phylum *Firmicutes*, whereas there were negative associations between 19 different *Lactobacillus* species and BP indexes (47). Importantly, the richness of opportunistic pathogenic taxa, gram-negative bacteria, and lactate-producing genera was increased, as well as the *Firmicutes/Bacteroidetes* ratio, which is deemed as an indicator of intestinal microbiota health (10, 27, 50, 51). However, as shown in Table 2, the studies have yielded inconsistent results, which may be largely due to the heterogeneity of intestinal microbiota. Existing evidence has demonstrated that fecal microbiota transplantation (FMT) from patients and animals with hypertension to normotensive individuals can elevate BP levels (41, 52), and impair endothelial function (53). Human and animal studies further indicated that the intestinal microbiota could reasonably be regarded as a fundamental mediator of hypertension. However, the optimal profile of the intestinal microbiota in animal models of hypertension or human subjects remains contentious or even inverse. Thus, clarifying the specific microbial alternations in the state of hypertension is the next urgent and challenging task.

**Table 2:** Composition of intestinal microbiota in patients with hypertension.

**Population**	**Detecting method**	**Main conclusions**		**References**
		**Higher abundance in the hypertension group**	**Higher abundance in the control group**	
4,672 subjects	16S ribosomal RNA sequencing	*Streptococcus, Klebsiella*	*Roseburia, Ruminococcaceae*	(39)
38 hypertensions, 7 borderlines, 9 controls	16S ribosomal RNA sequencing	*Clostridum sensu stricto 1*	*Ruminococcaceae, Clostridiales*	(37)
239 subjects	16S ribosomal RNA sequencing	*Collinsella, Actinobacteria, Bifidobacterium*	*Bacteroidetes, Alistipes genus*	(40)
99 hypertensions, 56 pre-hypertensions, 41 controls	Metagenomic sequencing	*Prevotella, Klebsiella, Desulfovibrio, Fusobacterium*	*Bacteroides, Butyrivibrio, Oscillibacter, Roseburia, Bifidobacterium, Coprococcus, Faecalibacterium, Clostridium*	(41)
529 subjects (183 hypertensions)	16S ribosomal RNA sequencing	*Anaerovorax, Clostridium IV, Oscillibacter, Catabacter, Robinsoneilla*	*Sporobacter, Ruminococcus, Akkermansia, Asaccharobacter*	(42)
67 hypertensions, 62 controls	16S ribosomal RNA sequencing	*Acetobacteroides, Alistipes, Bacteroides, Barnesiella, Butyricimonas, Christensenella, Clostridium sensu stricto, Cosenzaea, Desulfovibrio, Dialister, Eisenbergiella, Faecalitalea, Megasphaera, Microvirgula, Mitsuokella, Parabacteroides, Proteiniborus*, and *Terrisporobacter*	*Acidaminobacter, Adlercreutzia, Anaerotruncus, Asteroleplasma, Bulleidia, Cellulosilyticum, Coprobacter, Enterococcus, Enterorhabdus, Guggenheimella, Lactivibrio, Lactobacillus, Marvinbryantia, Olsenella, Paraprevotella, Parasutterella, Phascolarctobacterium, Prevotella, Romboutsia, Ruminococcus, Sporobacter, Sporobacterium, Sutterella, Vampirovibrio, Veillonella*, and *Victivallis*	(43)
22 hypertensions, 18 controls	Shotgun metagenomic analysis	*Parabacteroides johnsonii, Eubacterium siraeum, Alistipes finegoldii*	*Bacteroides thetaiotaomicron, butyrate-producing bacteria*	(44)
60 hypertensions, 60 controls	Whole-metagenome shotgun sequencing	*Klebsiella, Clostridium, Streptococcus, Parabacteroides, Eggerthella*, and *Salmonella*	*Faecalibacterium, Roseburia, and Synergistetes*	(45)
29 hypertensions, 32 controls	16S ribosomal RNA sequencing	*Bacteroides coprocola, Bacteroides plebeius, genera of Lachnospiraceae*	*Ruminococcaceae, Ruminococcaceae, Christensenellaceae, Faecalibacterium prausnitzii, Roseburia hominis*	(46)
6,953 subjects	Shotgun metagenomic analysis	*Firmicutes*	*Lactobacillus*	(47)

In addition, patients with hypertension also had lower α-diversity and abundance of short-chain fatty acids (SCFAs)-producing microbiota (10, 39, 50). SCFAs are saturated fatty acids that contain carbon chains of one to six carbons in length, the main SCFAs in the human body are acetate, propionate, and butyrate (54). SCFAs serve as energy substrates for intestinal epithelial cells as well as key regulators of anti-inflammatory responses, lipid metabolic pathways, and gluconeogenesis *via* a series of G-protein-coupled receptors (GPCRs) (55). Verhaar et al. studied the feces of 4,672 individuals (49.8 ± 11.7 years, 48% men) from six different ethnic groups, finding that *Roseburia* spp., *Clostridium* spp., *Romboutsia* spp., and *Ruminococcaceae* spp. were the best microbial predictors for SBP. Fecal SCFA levels, such as acetate and propionate, were lower in young Dutch participants with low SBP (39). Bier et al. observed a negative correlation between the taxa of the *Actinobacteria* phylum and the butyric acid level, independent of dietary changes in Dahl salt-sensitive rats (24). Lactate-producing bacteria are positively associated with SBP, whereas butyrate-producing bacteria and acetate-producing microbiota are negatively associated with SBP (56, 57).

## The key mechanisms of intestinal microbiota in regulating the development of hypertension

### Intestinal microbiota drives hypertension *via* the nervous system

**Figure 2:**
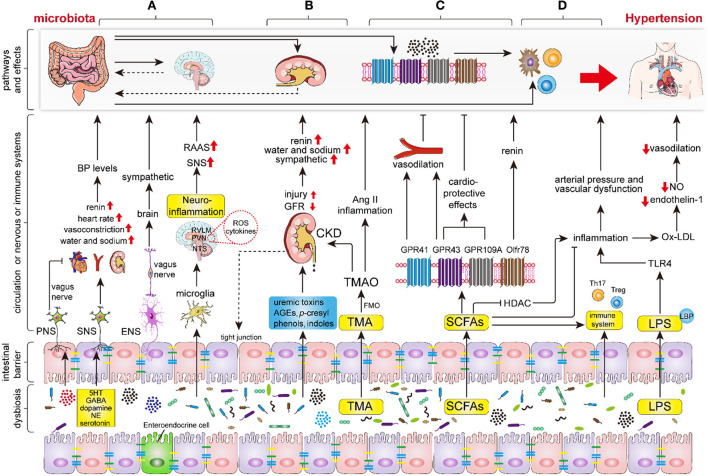
The key mechanisms of intestinal microbiota in regulating the hypertension process. Intestinal dysbiosis leads to disruption of the nervous system, renal function, intestinal SCFAs, immune system, and increases LPS production and intestinal permeability, thereby aggravating the progression of hypertension. **(A)** Intestinal microbiota and its metabolites (mainly including GABA, dopamine, noradrenaline and 5HT) regulate hypertension by modifying sympathetic and vagal nerve activity and neuroinflammatory response through the gut-brain axis. **(B)** Intestinal microbiota and its metabolites regulate hypertension *via* renal sympathetic regulation, humoral regulation, vasoactive substances (RAAS, nitric oxide, and endothelin), and even lead to hypertension by triggering chronic kidney disease (CKD). **(C)** The indigestible dietary fiber was fermented into SCFAs, primarily involving acetate, propionate, and butyrate, by intestinal microbiota. Subsequently, the SCFAs regulate hypertension *via* binding to the specific GPCRs which mainly include GPR43, GPR41, GPR109A, and Olfr78. **(D)** Intestinal microbiota and endotoxins (LPS) regulate hypertension *via* the immune and inflammation systems. Abbreviations: BP, blood pressure; PNS, peripheral nervous system; SNS, sympathetic nervous system; ENS, enteric nervous system; RAAS, renin-angiotensin-aldosterone system; GABA, gamma-aminobutyric acid; NE, noradrenaline; 5HT, 5-hydroxytryptamine; ROS, reactive oxygen species; TMA, trimethylamine; TMAO, trimethylamine-N-oxide; FMO, flavin monooxygenases; CKD, chronic kidney disease; SCFAs, short-chain fatty acids; GPR, G protein coupled receptor; HDAC, histone deacetylase; TLR4, Toll-like receptor 4.

As previous studies have shown that the sympathetic nervous system (SNS) modulates BP levels by promoting peripheral vasoconstriction, and heart rate, and by regulating water and sodium balance by innervating the nephron, the renal vasculature, and the juxtaglomerular cells (58–60). Excessive sympathetic activation was considered to be one of the major pathogenetic mechanisms of hypertension. Importantly, recent studies have demonstrated that sympathetic activation could be regulated by intestinal microbiota (Figure 2A). Studies have revealed that the microbiota modulates sympathetic activation *via* a gut–brain circuit mediated by the metabolites, including gamma-aminobutyric acid, dopamine, noradrenaline, and serotonin (5-hydroxytryptamine, 5HT), which are synthesized by intestinal microbiota, such as *Candida, Escherichia, Streptococcus, Bacillus*, and *Enterococcus* (56, 61, 62). Specifically, the dysbiosis-related bacterial metabolite imbalance increases the production of serotonin 5HT by the enterochromaffin cells in the gut (63); then, 5HT can modulate the activity of gut vagal afferents *via* 5HT3 receptors (5HT3Rs) potentially dampening the vagal gut–brain neural axis, whereas 5HT released into circulation can affect the vasculature and cause vasoconstriction (64). Nonetheless, the increased sympathetic activation can also contribute to epithelial dysfunction, increased intestinal permeability, and dysbiosis, increasing the translocation of microbiota metabolites into circulation (64). As a result, it further adversely affects the cardio-renal tissues.

Intestinal dysbiosis contributes to the increase of intestinal barrier permeability and activation of immune cells, which leads to impaired blood–brain barrier (BBB) function and directly results in central nervous system neuroinflammation, which plays a pivotal role in the progression of hypertension (16, 65). Furthermore, intestinal microbiota alteration affects the cerebral immune cells, such as microglia, the most abundant resident immune cells in the brain (66–68). Sharma et al. found that the inhibition of microglial activation in PVN inhibited sympathetic activation, and lowered BP in Ang II-induced rodent hypertension (22). Toral et al. demonstrated that the alteration of intestinal microbiota influences PVN NADPH oxidase activity, neuroinflammation, and sympathetic activity, subsequently impacting BP levels (57). The activated microglia release a variety of pro-inflammatory and toxic products, including ROS and cytokines (69), which indirectly promote sympathetic activation and increase BP (70). Erny et al. found that defective microglia can be repaired by microbiota or SCFAs (71). Meanwhile, intestinal dysbiosis can also promote Th1 cell infiltration, allowing local crosstalk with M1 microglia, which in turn triggers the differentiation of microglia to a pro-inflammatory state (68). Moreover, products of the intestinal microbiota, such as indoxyl sulfate, increase neuroinflammation (72). Intestinal dysbiosis also decreased levels of H2S, which is an endogenous vasoactive factor and a neuromodulatory and neuroprotective molecule that plays an antihypertensive and anti-neuroinflammatory role (70).

### Intestinal microbiota influences hypertension through the renal pathway

The kidney plays important roles in the pathogenesis of hypertension *via* renal sympathetic regulation, humoral regulation, and vasoactive substances (e.g., RAAS, nitric oxide, and endothelin). Recently, the intestinal microbiota has been identified as a substantial participant in regulating the progression of kidney disease through the gut–kidney axis (16). Intestinal dysbiosis contributes to the overproduction and accumulation of uremic toxins, *p*-cresyl, phenols, advanced glycation end products, and indoles, which all impair the intestinal barrier and increase intestinal permeability, and infiltrate the kidneys *via* the circulation, leading to the progression of kidney disease (73, 74). Moreover, the intestinal inflammatory cells and cytokines contribute to renal inflammation and injury *via* circulation (16).

In addition, L-carnitine, choline, and lecithin can be converted into trimethylamine (TMA) by intestinal microbiota, such as *Clostridia, Enterobacteriaceae, Anaerococcushydrogenalis*, and *Edwardsiella tarda* (75, 76). Subsequently, TMA was converted to trimethylamine-*N*-oxide (TMAO) by flavin monooxygenases (FMO) in the liver and excreted through the kidneys. Important Ang II-induced hypertensive mouse model showed a higher TMAO levels which were intimately associated with CVD and CKD (58, 77). Animal studies showed that increased TMAO in a long-term diet directly contributes to progressive renal fibrosis and dysfunction (75, 77) and the vasoconstriction of renal afferent arterioles (78). TMAO induces the production of pro-inflammatory cytokines, such as TNF-α and IL-1B, and inhibits the production of anti-inflammatory cytokines, such as IL-10 (79). Studies have disclosed that the circulating TMAO level was positively correlated with BP, which may be associated with endothelial dysfunction, oxidative stress (11, 80, 81), and the prolonged hypertensive effects of Ang II (82, 83). Moreover, the elevated level of TMAO was suggested as an increased risk for myocardial infarction, heart failure, peripheral artery disease, and stroke (10). Recently, Jaworska et al. demonstrated that TMA, but not TMAO, can affect the viability of human vascular smooth muscle cells, thereby exerting a booster effect of hypertension (84). Hong-Bao Li et al. demonstrated that the *Faecalibacterium* genus was significantly depleted in patients with CKD and hypertension (CKD-hypertension) compared with the healthy controls. The supplementation of *Faecalibacterium prausnitzii* to CKD mice reduced renal dysfunction and inflammation by the *Faecalibacterium prausnitzii*-induced butyrate–renal GPR-43 axis (85), whereas *F. prausnitzii* supplementation may alleviate BP in patients with CKD-induced hypertension.

### The impact of SCFAs produced by intestinal microbiota on hypertension

In the gut, indigestible dietary fiber is fermented into SCFAs, primarily involving acetate, propionate, and butyrate, by specific intestinal microbiota, including *Roseburia, Ruminococcaceae*, and *Faecalibacterium* spp (9, 14, 86); these intestinal microbiota have been demonstrated to be associated with moderate BP maintenance and shown to be less abundant in patients with hypertension (37, 44, 50, 87). In addition, the administration of a high-fiber diet and acetate supplementation showed significant alteration of the intestinal microbiota components, causing a remarkable increase in the proportion of *Bacteroides acidifaciens*, and led to a reduction in systolic BP (SBP) and diastolic BP (DBP) (3, 51).

Molecular mechanism studies have revealed that SCFAs regulate BP through a number of GPCRs, which mainly include the fatty acid receptor (FFAR)-2 (GPR43), FFAR 3 (GPR41), GPR109A (HCAR2), and olfactory receptor 78 (Olfr78) (9, 50, 62) (Figure 2). Animal studies showed that the activation of GPR41 in vascular endothelial cells stimulated by SCFAs leads to BP lowering and that mice lacking GPR42 (*Gpr*41^−/−^) have systolic hypertension, aortic thickening, and collagen deposition (88, 89). Similarly, GPR43 is widely expressed in a variety of tissues and plays a role in lowering BP in response to the stimulation of SCFAs (50). Waghulde et al. demonstrated that salt-sensitive hypertensive rats lack G protein-coupled estrogen receptor 1 (*Gper*1^−/−^), was accompanies the amelioration of hypertension, as well as the intestinal dysbiosis indicated by the diminished *Firmicutes*/*Bacteroidetes* ratio (90). Although the exact molecular mechanism by which the deletion of *Gper1* mitigates hypertension remains unclear, the researchers speculated that this process may be mediated by the gut microbiota (90). Olfr78 is localized in the renal afferent arteriole and vascular smooth muscle cells. In response, SCFAs, which mainly contain acetate and propionate, perform a unique role in facilitating renin secretion, eventually leading to high renin concentration and hypertension (50, 91, 92). Moreover, Olfr78 activation induced a counteraction of the hypotensive effect induced by GPR41 (93).

SCFAs are regarded as an important link between the intestinal microbiota and the immune system (3, 94), and selectively support the development of Th1, Th17, and Treg cells according to the cytokine and immune milieu (95). Studies revealed that SCFAs, particularly butyrate, mediate anti-inflammatory effects by inhibiting histone deacetylase (HDAC), and the inhibition of HDAC reduces pro-inflammatory and hypertensive responses by decreasing the production of ROS and the expression of Ang II type1 receptor (AT1r) in the myocardium (96, 97). Recently, Robles-Vera et al. demonstrated that acetate or butyrate supplementation prevented the development of hypertension in SHR, and restored Th17/Treg balance in the aorta (98). Furthermore, SCFAs can dampen glial inflammatory responses and subsequently decrease BP (70). Studies have demonstrated that SCFAs and FFA2 receptors improve the development of hypertension by inhibiting monocyte and dendritic cell activity (99).

In addition, vagal afferent receptors express SCFAs sensing to participate in BP modulation. SCFAs have also been suspected to participate in gut–brain communication, thereby becoming involved in the neural regulation of BP. FMT from normotensive to hypertensive animals has been shown to ameliorate BP levels in hypertensive animals and was accompanied by increased expression of GPR41 and GPR43 in PVN (64). Yang et al. showed that excessive colonic acetate levels can lower BP by activating parasympathetic nerves. Meanwhile, the diminished expression of butyrate-sensing receptors in the hypothalamus contributed to destroying the BP alleviation induced by butyrate administration in SHR (100). Therefore, the reduction in the availability of circulating SCFAs and SCFA-sensing receptors contributes to the pathophysiology of hypertension.

### Intestinal microbiota regulates hypertension mediated by the immune and inflammation system

The immune system and exaggerated inflammation have been demonstrated to play important roles in the hypertension process (101–103). Recently, the intestinal microbiota was indicated as a key regulator of the immune and inflammatory response (104) (Figure 2). The intestine is the largest immune organ in the body, with a complex mucosal immune system, lymphocytes, and innate immune cells spread throughout the epithelial layer (16). Moreover, the development of the intestinal immune system depends on the intestinal microbiota (105). The lack of intestinal microbiota can lead to inadequate gut-associated lymphoid tissues (GALTs) development and systemic and central immune abnormalities (16, 106, 107). Germ-free animals are accompanied by a significant reduction in the richness of TH17 cells, B cells, and disequilibrium of TH1 and TH2 responses, and impaired Treg cell function (16). Preclinical studies have shown that T lymphocyte subsets, such as Th1, Th2, Th17, and Treg cells, participate in the regulation of BP and end-organ damage (3, 108). Th1 and Th17 cells release pro-inflammatory cytokines, such as IFN-γ, TNF-α, and IL-17a, which are prominent pathogens in hypertension models (104, 109) while Treg cell inhibits Ang II-induced hypertension by releasing anti-inflammatory cytokines, which mainly include IL-10 and TGF-β (110).

The intestinal dysbiosis, including the increased *Bilophila wadsworthia* and *Clostridium cocleatum* and the decreased *Bifidobacterium* and *Bacteroides*, leads to an increase in LPS production and permeability of the intestine (111). LPS infiltrates into the circulation across the highly permeable intestinal wall to form a complex with LBP, which binds to CD14 on monocytes, contributing to the production of pro-inflammatory cytokines, such as TNF-α, IL-1, and IL-6 (50, 112). Additionally, LPS also interacts with PRR (TLR4) to induce inflammatory responses and increase arterial pressure and vascular dysfunction (113–116). In animal models, LPS administration caused an increase in heart rate, norepinephrine level, and neuroinflammation, as well as decreased baroreflex sensitivity, which was confirmed by increased expression of TLR and TNF-α in the PVN (117). Wang et al. discovered that the inhibition of TLR4 in PVN caused amelioration of hypertension in SHR *via* a decrease in ROS production and pro-inflammation cytokines (118). Moreover, intestinal dysbiosis also directly increases the production of pro-inflammatory cytokines and ROS, which leads to the production of ox-LDL (13). Subsequently, Ox-LDL has an inhibitory function in the production of nitric oxide (NO), a well-recognized vasodilator, and endothelin-1, leading to the exacerbation of hypertension (119). The intestinal microbiota at least partially contributes to Ang II-induced vascular dysfunction and hypertension by supporting MPC-1/IL-17-driven vascular immune cell infiltration and inflammation (120). SNS directly innervates the primary immune organ (bone marrow), and sympathetic activation can induce bone marrow hemopoietic stem cells into a pro-inflammatory state through the brain–gut–bone marrow axis (121, 122). Bone marrow-derived immune cells can be activated by the intestinal microbiota, leading to low-grade chronic inflammation, which is a recognized risk factor for hypertension (16, 101).

## The therapeutic potential for targeting intestinal microbiota in hypertension

Recent evidence suggests that intestinal microbiota-related strategies, such as FMT, probiotics, prebiotics, and synbiotics, may be considered as promising strategies for the prevention and treatment of hypertension (10, 123, 124). In high-salt-induced hypertensive mice, FMT administration decreased the richness of *Allobaculum, Dubosiella*, and *Alloprevotella*, but increased the relative abundance of *Lachnospiraceae_UCG-006* and *Lachnoclostridium* (125). Adnan et al. transplanted the fecal microbiota extracted from spontaneously hypertensive stroke-prone rats into WKY rats and found an increase of 26 mmHg in SBP in WKY rats, accompanied by a significantly increased ratio of *Firmicutes/Bacteroidetes* (28). Importantly, the transplantation of microbiota from normotensive into hypertensive animals has caused the amelioration of BP levels in animal models of hypertension and was accompanied by an increase of SCFAs receptors expression in PVN (64). Toral et al. found that FMT from WKY rats to SHR rats decreased basal SBP, restored the Th17/Treg imbalance, improved endothelial dysfunction, and alleviated vascular oxidative stress (53). Unfortunately, no studies have been published on the treatment of hypertension with FMT.

Probiotics, containing SCFAs-producing microbiota, are defined as living microorganisms that can modify the composition of the microbiome to benefit the host (11, 15). Probiotics supplementation is conducive to intestinal barrier function, reduces endotoxemia and increases butyrate (15, 98), and further significantly lowers SBP and DBP (49). Robles-Vera et al. demonstrated that probiotic *Bifidobacterium breve* CECT7263 (BFM) prevented DOCA-salt hypertension and renal damage by increasing acetate and reducing TMA production (23). Similarly, Wilck et al. found that salt-sensitive mice supplemented with *Lactobacillus murinus* had ameliorated hypertension compared with controls (25, 126). Nonetheless, the antihypertensive activity of probiotics depends on the specific strain; even for the same strain of probiotics, the antihypertensive effect varies in different hypertension models (49). Intervention with *Lactiplantibacillus plantarum* strains SR37-3 (PFM-SR37-3) and SR61-2 (PFM-SR61-2) significantly lowered the BP of NG-nitro-L-arginine methyl ester induced hypertensive rats and attenuated renal injury (127).

Prebiotics are defined as a healthy matrix that is selectively utilized by host microorganisms to promote the growth of beneficial intestinal microorganisms (128, 129). Kaye et al. found that the administration of prebiotic fiber has protective effects on hypertension and cardiac hypertrophy, mediated by GPR43/GPR109A (9). Hsu et al. reported that the prebiotic treatment prevented BP elevation and diminished the ratio of *Firmicutes*/*Bacteroidetes* (130). Meanwhile, the renal mRNA expression of ACE and plasma TMAO levels were concomitantly decreased (130).

The word synbiotics refers to a mixture of living microorganisms and substrates that are selectively utilized by the host microorganisms and beneficial to the host's health (131). A systematic review and meta-analysis of clinical trials conducted by Hadi et al. showed that synbiotic interventions improved SBP in patients with hypertension (128). Similarly, Bartolomaeus et al. showed that synbiotic management increased intestinal SCFAs production and significantly reduced BP levels in patients (132).

## Conclusion and perspective

Hypertension presents a significant public health challenge and is a major risk factor for CVD, cerebrovascular, and CKD (133). Accumulating evidence in recent years supports that the intestinal microbiota and its metabolites are essential regulators of hypertension and its complications (11). Alterations in the intestinal microbial composition associated with disease and potential virulent metabolites have been considered a contributor to the development of hypertension. Sympathetic activation and neuroinflammation, induced by intestinal dysbiosis, have been recognized as key players in hypertension. Similarly, intestinal microbial metabolism, such as SCFAs, TMAO, and endotoxins (LPS), has a substantial influence on hypertension through its effect on undulating vasomotion, renal function, neural activation, and inflammation.

Consequently, modification of the intestinal microbiota is considered a promising tactic to improve personalized BP control. Nevertheless, owing to past technical limitations and insufficient understanding of intestinal microbiota, the current in-depth research on intestinal microbiota and hypertension remains limited. Currently, evidence in humans is circumspect and indeterminate, and the understanding of the mechanisms is widely based on rodent models. In many cases, the causal relationship between dysbiosis and hypertension is still inconclusive. In addition, the specific mechanism by which intestinal microbiota affects the progression of hypertension remains largely ambiguous. The lack of longitudinal studies makes it extremely challenging to identify the specific alternations in the intestinal microbiota of patients with hypertension; hence, individualized treatment consisting of modulation of the microbiota remains largely challenging. Furthermore, the safety and effectiveness of preventing and treating hypertension *via* intervention in the intestinal microbiota require further evaluation. Therefore, the management of the intestinal microbiome for the prevention and treatment of patients with hypertension is full of prospective challenges and potential.

In the future, genome-wide correlation studies should be combined with intestinal microbiome analysis to provide personalized data on the individual composition of the microbiota. The mechanism through which the intestinal microbiota affects hypertension requires further investigation, particularly regarding the specific bacterial species. In addition, more clinical evidence is required to validate the results of experiments in rodent models. It is extremely important to emphasize that the main site for sensing pro-hypertensive signals is the brain or the intestine, or both contribute equally.

In conclusion, the implementation of individualized intestinal microbiota intervention therapy strategies for hypertension is expected to occur. The use of intestinal microbiota modulation as a therapeutic option for hypertension has excellent potential in the development of personalized strategies for hypertension management.

## Author contributions

LH and JY contributed to the conception and design of the study. DS and HX wrote the first draft of the manuscript. All authors reviewed and edited the manuscript. All authors contributed to the article and approved the submitted version.

## Funding

This work was supported by Wuhan No. 1 Hospital, Wuhan Hospital of Traditional Chinese and Western Medicine.

## Conflict of interest

The authors declare that the research was conducted in the absence of any commercial or financial relationships that could be construed as a potential conflict of interest.

## Publisher's note

All claims expressed in this article are solely those of the authors and do not necessarily represent those of their affiliated organizations, or those of the publisher, the editors and the reviewers. Any product that may be evaluated in this article, or claim that may be made by its manufacturer, is not guaranteed or endorsed by the publisher.
